# Clinical Psychology Services for Patients Hospitalized Due to COVID-19 During the Pandemic in Northern Italy: From Isolation to Rehabilitation

**DOI:** 10.3389/fpsyg.2021.588193

**Published:** 2021-03-26

**Authors:** Edward Callus, Enrico Giuseppe Bertoldo, Valentina Fiolo, Silvana Pagliuca, Barbara Baroni

**Affiliations:** ^1^Department of Biomedical Sciences for Health, Faculty of Medicine and Surgery, University of Milan, Milan, Italy; ^2^Clinical Psychology Service, IRCCS Policlinico San Donato, San Donato Milanese, Italy; ^3^Orthopedic Rehabilitation Operative Unit, IRCCS Policlinico San Donato, San Donato Milanese, Italy

**Keywords:** COVID-19, clinical psychology, telehealth, anxiety, depression, PTSD, sleep disorders, rehabilitation

## Abstract

The objective of this paper is to describe the organization and modality of provision of clinical psychology services for those patients who had to be hospitalized due to COVID-19 during the pandemic in Northern Italy. The IRCCS Policlinico San Donato hospital in Milan was converted into a COVID-19 center in March 2020, and all the staff, including the Clinical Psychology Service Team, were diverted to assist these patients. A description is given of how the service was organized and the modalities which were utilized to assist the patients. Following the pertinent ministerial decrees, guidelines, and relevant literature, the patients were followed up through telehealth (via phone, smartphone, or tablet with audio or audio-visual calls). A COVID-19 rehabilitation unit was later opened in April 2020, where all patients were seen and followed up by the Clinical Psychology team, the last patient being discharged at the end of June. Details are given about the type of services provided during the hospitalization at the different points in time. At admittance and subsequent isolation, patients indicated by the medical and nursing staff, and those who specifically requested it, were given psychological support. Patients transferred to the COVID-19 rehabilitation unit were all evaluated for anxiety, depression, posttraumatic stress disorder, and sleep disorders both on admission and at discharge when possible.

## Introduction: The Outbreak of COVID-19 in Northern Italy

The official date of the start of the outbreak of COVID-19 in Italy was the 31st of January 2020. This followed the arrival of two Chinese visitors on a flight from Wuhan to Milan Malpensa Airport on the 23rd of January, who tested positive in a central hotel in Rome. The first Italian patient confirmed with COVID-19 (patient 1) was a 38-year-old man admitted to the hospital in Codogno, Lombardy, on the 21st of February. After that, in <1 week there was a surprising increase in the number of cases in Italy, which put the Italian health service under considerable strain, leading to the discovery of cases in various bordering regions and autonomous provinces of northern Italy (Carinci, 2020).

Following these events, many hospitals in northern Italy were converted into COVID-19 centers to deal with the emergency. On the 9th of March, it was communicated that also the IRCCS Policlinico San Donato would become a COVID-19 center and that all the staff, including the Clinical Psychology Service Team, would need to be diverted to assist this population. This caused considerable strain on the team, which had to quickly get organized to provide psychological assistance to a new population by utilizing new modalities. There was the additional stress of finding oneself in the same situation of the patients and the families themselves (The British Psychological Society, 2021), which was handled by holding daily remote meetings. During the peak of the pandemic, 283 hospital beds were made available for COVID-19 patients in our institute, and a total of 632 COVID-19 patients were admitted to our center, until the 30th of April. After that date, some patients were followed remotely after their discharge if required and but no further COVID-19 patients were admitted.

## Psychological Factors Associated With Hospitalization of COVID-19 Patients

In the COVID-19 Prevention and Treatment Manual 2020a, it is reported that in isolation wards about 48% of confirmed COVID-19 patients experience psychological stress during initial admission.

The organization of health care for COVID-19 patients must necessarily include mental health care in a timely and urgent manner (Bao et al., 2020; Carvalho et al., 2020; Jiang et al., 2020; Onyeaka et al., 2020; Xiang et al., 2020).

Specific attention needs to be given to the older population, who are considered more vulnerable and more limited in regard to technological access and to remote care (Yang et al., 2020).

In fact, anxiety, and sleep disturbances have been shown to increase significantly following isolation hospital-based treatment. However, preliminary findings indicate that asymptotic muscle relaxation training, also conducted remotely, can be used to alleviate anxiety and improve sleep quality of patients with COVID-19 (Liu et al., 2020a).

In this study based in China, quality of life, anxiety, and depression were specifically assessed in the rehabilitation setting, and both quality of life and anxiety improved after a 6-week program (Liu et al., 2020b) in which the following interventions were carried out: respiratory muscle training, cough exercise, diaphragmatic training, stretching exercise, and home exercise.

Practitioners working in respiratory rehabilitation can also deal with reducing anxiety and depression in patients who experience symptoms of deliriousness, anger, fear, dysthymia, insomnia, panic attacks, or sense of abandonment during isolation and to monitor and improve the quality of life as much as possible. In addition, it is important to monitor the possibility of non-collaboration and non-adherence to treatment (Vitacca et al., 2020; Zhou et al., 2020). From our clinical experience, non-adherence to treatment in hospital may occur because the patients are not aware of the effects of the treatment, because of extreme fatigue and because of severe psychological distress. Psychologists can help patients understand the importance of their treatment and provide support and indications for relaxation.

## National and International Guidelines and Recommendations About Psychological Support During the COVID-19 Pandemic

As a consequence of the pandemic, a series of decrees were issued by the President of the Council of Ministers in Italy *(Presidente del Consiglio dei Ministri – PDCM)* 2020b. In particular, on the 8th of March 2020 a decree was issued (PCDM, 2020) in which it was specified that in order to “counter and contain the spread of the COVID-19 virus,” “movements are authorized only if motivated by work needs or situations of need, that is displacement for health reasons.” It was highlighted that in all possible cases, “remote connection modalities with particular reference to public and community health and public utility services” must be adopted, to find alternative ways to proceed rather than physically moving people between locations.

Interim guidelines that addressed the use of personal protective equipment with coronavirus disease (COVID-19) and for operating during periods of severe shortages (World Health Organization, 2020), indicated that all bedside contact between health workers and COVID-19 patients should be restricted to only staff providing direct care, and that even this contact should be minimized and carefully planned. The advice to psychologists with respect to COVID-19 issued by the National Council of the Order of Psychologists (*Consiglio Nazionale Ordine Psicologi - CNOP)* (Consiglio Nazionale Ordine Psicologi, 2020) recommended that consultancies and therapies take place remotely when faced with clear epidemiological risk factors. The relevant regional order of psychologists recommended that the guidelines specified in the ministerial decree issued on the 8th of March should also be followed by psychologists in relation to organizing their work (Ordine Degli Psicologi Della Lombardia, 2020).

Further, international research and guidance papers recommended that psychological services should be delivered remotely, online, and/or over the telephone (Carvalho et al., 2020; Jiang et al., 2020; Liu et al., 2020c; Wind et al., 2020; Wright and Caudill, 2020; Xiang et al., 2020; Zhou et al., 2020). Nonessential staff, such as psychiatrists, psychologists, and social workers, were strongly discouraged from entering isolation wards for COVID-19 patients to observe the strict infection measures required by the pandemic (Duan and Zhu, 2020). It was suggested that patients should be guided to complete relevant questionnaires via their mobile phones 2020a.

In the recommendations relating to respiratory rehabilitation, it was indicated that psychological support is also important at the time of hospitalization (Brugliera et al., 2020; Xiao et al., 2020). Further, rehabilitation programs for those patients who have been isolated should be conducted remotely with telehealth systems, in order to avoid the spread of infection (Vitacca et al., 2020; Zhou et al., 2020).

## Organization and Modality of Delivery of Clinical Psychology Services

The rapid evolution of the situation in Northern Italy did not allow the possibility of structuring an intervention plan. In fact, before our institute was converted to a COVID-19 center in 2020, all of the Clinical Psychology Service staff were exposed to the virus without any personal protective equipment, having been in the presence of patients who were later diagnosed as having a positive diagnosis. All team members tested negative at the COVID-19 screening. However, in May, after a period of quarantine that followed this initial exposure, one psychologist's results indicated the presence of virus antibodies (but not the virus itself).

In order to establish how to proceed, a literature review was carried out. The pertinent ministerial decrees and indications from the regional order of psychologists were consulted, which will be described in detail in the following paragraphs. Operative instructions were drafted based on the decrees and guidelines and inserted in the official system of the hospital. These were automatically forwarded to all the staff of the hospital via e-mail, initially to provide guidance for the care of patients hospitalized for acute admissions. These were subsequently also applied for patients in rehabilitation following the opening of the COVID-19 Rehabilitation Unit on the 6th of April 2020.

In both cases, the operative instructions describe the expected psychological pathway for patients hospitalized in the various departments and therefore urgently requiring psychological crisis intervention (Jiang et al., 2020; Parolin et al., 2020).

## Modalities of Delivery of Psychological Support in the Various Operative Units at the IRCCS Policlinico San Donato Hospital

Taking into consideration all guidelines and recommendations, all clinical psychology services were organized online and/or via telephone. In those cases when patients did not own a smartphone or mobile phone, arrangements were made with staff to use the departmental telephone or tablet.

### Patients in Isolation and Intensive Care Unit

During the pandemic, more than 283 hospital beds (out of which 28 were intensive care beds) were dedicated to COVID-19 patients. For those patients who were in isolation and hospitalized in the intensive care unit. Although psychological support was provided for patients in the intensive care unit, no psychometric evaluations were carried out, in order to avoid the risk of contributing further to their distress. Due to the large number of patients hospitalized, it was not possible to contact all of them to enquire whether or not they required psychological support.

The main route of referrals to the Clinical Psychology Service was through the medical and nursing staff, who alerted other colleagues via e-mail, phone, and word of mouth that the service was available remotely when required. A WhatsApp message was also circulated, instructing the medical staff on the procedure for referring patients.

The medical and nursing staff could refer the patients and their relatives by calling the service's fixed line from 8 a.m. to 8 p.m. (7 days a week), by writing an e-mail, or by messaging or calling the Clinical Psychology Service team mobile phone numbers.

The following information was requested when referrals were made: first and last name of the patient, their age, a brief description of their medical situation, the telephone number on which the patient could be contacted (possibly specifying if it is a smartphone or a normal phone), the operative unit in which the patient was hospitalized, and the phone contact of the referring physician and the head nurse of the operative unit.

During this phase, the type of psychological intervention was based upon the evaluation which was carried out during the first session, taking into consideration the presence of psychological distress (including anxiety, depression, and sleep disturbance), the patients' psychosocial situation, and the support they currently feel they had. All the questionnaires utilized have cutoffs which indicate the level of distress. When patients reported moderate or severe distress, this was explored and the patients were closely monitored, on a daily basis. If the distress was absent or mild, the patients were contacted once or twice a week.

A total of 21 patients were seen (9 females and 12 males, average age 67 years, ages ranging from 31 to 84 years) from the 9th of March to the 6th of June. The average duration of hospitalization was 25 days, and 189 sessions were provided, which lasted approximately 20 min each. 13 family members were also supported, and 2 patients were also provided psychological support after discharge. One patient who was referred to the Clinical Psychology Service refused the offer of support.

### Patients in COVID-19 Rehabilitation Unit

When it comes to performing assessments in COVID-19 rehabilitations, it has been recommended that questionnaires which can be compiled quickly by patients, and which can help to quickly identify the type of psychological dysfunction should be utilized (Zhao et al., 2020). In addition, patients should have access to psychological support also through a hotline (Zhao et al., 2020). Further, it is recommended to provide patients counseling sessions, psychological support, and cognitive training as required (Brugliera et al., 2020).

When the patients were transferred to the rehabilitation unit, they were in a more stable condition, and this allowed for a more thorough psychological screening with the utilization of psychometric tests and a psychosocial assessment. The average length of hospitalization in this unit was of 18 days (with a minimum of 8 days and a maximum of 39 days).

The patients were identified upon hospitalization in the unit by the communication of the medical staff to the clinical psychology service staff and through the daily consultation of the GALILEO, a management system for coordinated activities to efficiently direct and govern patient-centered hospital processes. The same information was obtained for patients in isolation if this had not previously been gathered.

For each patient, a minimum of 3 sessions were programmed during their hospitalization of about 20 days; if the patient stayed for <10 days, at least 2 sessions were provided. At the end of each session, an activity report file which included the date, the starting time of the end time of the session, the duration in minutes, and the qualitative description of the type of intervention was compiled by the staff of the service on an online excel database.

During the first interview, a general assessment of the patients' psychological status was carried out as well as the appropriateness of administering psychometric tests.

In the second session, if the psychosocial questionnaire could be administered to the patient, the assessment was carried out remotely. If, for some reason, it was not possible to administer the psychosocial questionnaire to the patient, a psychological support session was provided. Some reasons which did not allow patients to be tested were the patients feeling too weak and tired, being completely deaf, severe cognitive impairment, and when patients refused to proceed.

When it comes to the third interview, if the hospitalization lasted <15 days, the psychometric tests were not re-administered (because most tests assess psychological functioning on a period of 2 weeks). If the patients' hospital admission lasted for 15 days or more, the psychometric tests were re-administered [anxiety (GAD-7), depression (PHQ-9), post-traumatic stress disorder (IES-R), and insomnia (ISI)], and the evolution of the psychological situation was discussed with patients.

For all patients entering the rehabilitation unit, two reports were drawn up: an initial psychological report, which included all the results from the psychometric tests, and the task report form, where all the sessions and the number of minutes were indicated. For patients who were hospitalized for 15 days or longer, a final psychological report was drafted, where the evolution of the psychological condition was described. When patients required and/or requested psychological support also after discharge, it was provided. All the documentation was sent via e-mail to the head of the COVID-19 Rehabilitation Unit via the hospital e-mail, to be included in the patients' medical records, and stored digitally in the area of the server dedicated to the service of Clinical Psychology.

A total of 37 patients were admitted to the COVID-19 Rehabilitation unit, and 35 were evaluated and provided support (18 females and 17 males, average age 75, age ranging from 52 to 92) from the 8th of April to the 30th of June. The remaining 2 patients could not be supported because one was completely deaf and the other one had severe Alzheimer's disease. Five of the patients who were supported during isolation were subsequently moved to rehabilitation. A total of 108 sessions were provided which lasted on average 20 min.

### Psychosocial Assessments Utilized in COVID-19 Rehabilitation

The following questionnaires which have been validated and freely distributed in Italy are suggested in order to monitor anxiety and depression: the Patient Health Questionnaire-9 (PHQ-9) (Spitzer et al., 1999; Cannon et al., 2007; Stafford et al., 2007; Kroenke et al., 2009) and the GAD-7-General Anxiety Disorder-7 (Spitzer et al., 2006; Esser et al., 2018). As specified in the previous paragraphs, it is extremely important to monitor anxiety and depression in hospitalized patients with COVID-19. The following short questionnaires may be particularly useful for measuring a range of psychological symptoms in this population.

The Impact of Event Scale (IES) (Spitzer et al., 2006; Esser et al., 2018) has been previously utilized to assess levels of posttraumatic stress disorder in patients infected with severe coronaviruses and who were also hospitalized. The initial assessment was carried out through a psychosocial questionnaire that evaluated the general patient history including demographic and social variables: marital status, presence/absence of children, culture of origin, education level, occupation status, and religion.

In regard to assessment of social support and loneliness, patients were asked if they had any close relationships, the quality of those relationships, and if they were receiving adequate support.

### Psychometric Testing

The following validated tests were administered after the psychosocial assessment.

- The Fagerström test (Heatherton et al., 1991) is a self-administered test that evaluates the current dependence on nicotine.
- The Morisky, Green, and Levine Adherence Scale (MGL) (Morisky et al., 1986) is the most widely used on adherence to drug therapy.
- The Satisfaction with Life Scale (SWLS) (Broadbent et al., 2006) measures the overall cognitive assessment of life satisfaction experienced by the patient.
- The Patient Health Questionnaire - 9 (PHQ-9): (c Kroenke et al., 2001) is a short psychological screening tool designed to measure symptoms of depression in primary care facilities.
- The Generalized Anxiety Disorder (GAD-7): (Kroenke et al., 2007) is a screening tool for generalized anxiety disorder in clinical practice and research. In addition, it provides a measure of severity and is linked to the criteria of the Diagnostic and Statistical Manual of Mental Disorders (DSM-IV).
- The EuroQoL-Visual Analog Scale (VAS): (EuroQol Group, 1990) is the second part of the EuroQoL-5D questionnaire, a standardized tool that measures the health of respondents and their quality of life and consists of a VAS of 20 cm, graphically represented as a graduated thermometer, on which the patient indicates the best (score−0) or worst (score−100) possible state of health.
- The QoL-VAS: (Moons et al., 2006) consists of a visual analog scale of 20 cm, graphically represented as a graduated thermometer, on which the patient indicates the best (score−100) or the worst (score−0) about the perception of own quality of life.
- The Impact of Event Scale (IES-R): (Weiss et al., 1997) is a self-report measure (for DSM-IV) that assesses subjective distress caused by traumatic events.
- The Insomnia Severity Index (ISI): (Bastien et al., 2001; Castronovo et al., 2016) is a self-report questionnaire assessing the nature, severity, and impact of insomnia.

If the cutoffs of the various tests indicated the presence of anxiety, depression, PTSD, and sleep disturbances and if there was a particular indication of social isolation and/or also a lack of awareness and adequate coping, the medical staff were alerted. These elements guided the psychologist to plan additional sessions as needed.

## Discussion and Conclusions

In this perspective paper, the organization of clinical psychology services for COVID-19 patients who were severe enough to necessitate hospitalization, in a red zone, was described. The progression of the pandemic was extremely rapid and unexpected in Northern Italy, and in that period, there was a lot of uncertainty about the characteristics of the virus. This caused a lot of difficulty when it came to the determination of the modality of the provision of the clinical psychological service and on the type of intervention which was required. Another peculiar characteristic, which varied from the routine situation, was that all the members of the staff were exposed to the same situation in which the patients were, even though none of them developed any severe symptoms.

The first cases which were referred to the service included young people who were affected with a Cytokine Storm. As the isolation departments were being organized, the medical staff requested our intervention. In the beginning, there was a lot of uncertainty about whether to proceed remotely or in person.

Following consultation of all the decrees, recommendations, and pertinent literature, there was no doubt that the interventions needed to be provided remotely, to minimize, as much as possible, the spread of the virus during the peak.

As indicated in the literature described in the previous paragraphs, in the absence of the option of in-person contact, that remote modalities remote psychological support is highly effective when it comes to the assessment and monitoring of the hospitalized patients and also the delivery of psychological support. Patients who had to wear breathing caskets because of a very low saturation confirmed that it was easier to communicate remotely, because they had the possibility to put earphones inside the casket. Face-to-face communication was extremely difficult also because the healthcare providers who were present had to wear a considerable amount of protective gear, making communication even more difficult.

During the sessions, the patients were helped to become aware of their emotions and express their distress (also with the help of the patient reported outcomes) and they were taught to relax through very basic breathing exercises. Their psychological status was monitored, and if at discharge they still reported experiencing distress, they were supported also during the follow up period. In agreement with the literature we cited, we therefore recommend providing remote psychological support to the patients and their families.

In order to monitor the well-being of Clinical Psychology Staff, a remote “check-up” was organized twice a day, once in the morning and once in the evening. Temperature and symptoms were checked, and support was provided also within the team, as required, to increase resiliency during this highly stressful period marked by significant uncertainty. In conclusion, even though patients in rehabilitation were all screened and supported, and all referred patients were contacted, it could be possible due to the highly demanding and unexpected circumstances that not all isolated patients who required psychological support were referred to and contacted by the Clinical Psychology Service.

We recommend that in future, it would be useful to identify medical and psychosocial characteristics that could help the clinical psychology service to recognize suitable patients independently, without the need for direct referrals by medical staff. Ensuring that patient mobile phone numbers are reliably recorded on their medical chart would facilitate this. Based on our experience, we suggest that indicators could be based on certain medical and psychosocial indicators (such as preexisting psychiatric or psychological conditions, possibility of a bad prognosis, etc.). Such measures as these may increase the efficiency and expediency of the provision of clinical psychology services in the midst of pandemic conditions.

## Data Availability Statement

The original contributions presented in the study are included in the article/supplementary material, further inquiries can be directed to the corresponding authors.

## Author Contributions

EC, SP, VF, and EB made substantial contributions in the conception, writing, and final approval of the article. BB reviewed the final version of the article.

## Conflict of Interest

The authors declare that the research was conducted in the absence of any commercial or financial relationships that could be construed as a potential conflict of interest.
